# Iron, folate and vitamin B_12_ status of Ethiopian professional runners

**DOI:** 10.1186/s12986-015-0056-8

**Published:** 2015-12-30

**Authors:** Kifle Habte, Abdulaziz Adish, Dilnesaw Zerfu, Aweke Kebede, Tibebu Moges, Biniyam Tesfaye, Feyissa Challa, Kaleab Baye

**Affiliations:** Food Science and Nutrition Research Directorate, Ethiopian Public Health Institute, Addis Ababa, Ethiopia; Center for Food Science and Nutrition, Addis Ababa University, Addis Ababa, Ethiopia; Micro Nutrient Initiative, Addis Ababa, Ethiopia

**Keywords:** Ethiopia, RBC, Ferritin, Micro nutrient deficiency, Food frequency, Dietary diversity, Runners

## Abstract

**Background:**

Better macro and micro nutrient status and their adequate intake by the athletes have great role in balancing losses associated with strenuous exercise, then for better performance. The objective of this study was to determine iron, folate and vitamin B_12_ status of Ethiopian professional athletes.

**Methods:**

A cross sectional study was conducted using a point time convenient sample of 101 male and female Ethiopian professional athletes of different distance categories in the period of February to April 2014. Biochemical samples, detail health and exercise related interview, performance data, 24 h dietary diversity and weekly food frequency were collected.

**Results:**

The low, medium and high dietary diversity terciles were 36.1, 60.9 and 3.3 % respectively. The mean ± Sd of dietary diversity was 5.44 ± 1.8. Prevalence of iron overload (Serum ferritin >200 μg/L) was 11 %, whereas that of anemia (Hb < 12 g/dL), iron deficiency (ferritin < 12 μg/L) and moderate folate deficiency (<5.9 ng/mL) was 3, 2 and 20.8 % respectively. There was no iron deficiency anemia case in the study. In this study, the mean serum vitamin B_12_ concentration was 561 ± 231 pg/ml with a minimum and maximum value of 210 and 1736 pg/ml respectively, and there was no deficiency for this nutrient (>210 pg/ml). The iron status of male athletes was significantly different by running-distance categories. In contrast, such difference was absent for female athletes. Performance of the athletes was associated with their red blood cell count (RBC) at *p* = 0.03. The high performer athletes exhibited high mean value of micronutrient status and hematological variables than their counter parts. However, the RBC of the athletes was the only parameter whose association was statistically significant.

**Conclusions:**

The observed gender difference in the association of running-distance category with iron and folate in this study needs further investigation. Given the 11 % iron overload in the present study; there is a need of awarance creation activities and diet intervention in the athletics federation, the athletes and the coaches in order not aggravate the present overload. Prescription of supplements such as iron-folate, multivitamins and minerals should not be based on broad spectrum rather it should be based on recent history of confirmed deficiency, clinical signs and/or laboratory testing to prevent trace element toxicity.

## Background

Almost One-third of world populations suffer from micronutrient deficiencies [1]. The most common micronutrient deficiencies include vitamin A, iron, iodine, vitamin B_12_, zinc and folic acid. The cause of these micro nutrient deficiencies is primarily due to prolonged inadequate dietary intake of foods rich in these micronutrients [2]**.**

Iron deficiency anemia (IDA) is the most prevalent micronutrient deficiency in the world and around two billion people are affected. It occurs in developing countries at greater rates [3]. There is no sufficient data yet for the rest of micronutrient prevalence such as folate and vitamin B_12_ [4]. Anemia is the decrease in number and size of red blood cells (RBCs) or decreased amount of hemoglobin (Hb) in RBCs. Iron deficiency (ID) occur when serum ferritin level below the normal range, that is below 15 μg/L [5].

Anemia and ID affects oxygen and carbon dioxide exchange between blood and tissue cells thus limits a person’s endurance and performance; because of this iron is an important nutrient in sports that require endurance for success [6, 7]. Many micronutrients play key roles in energy metabolism and during strenuous physical activity, the rate of energy turnover in skeletal muscle can be increased up to 20–100 times the resting rate. Marginal deficiency states may have no great impact on the sedentary individuals, but may have extreme consequences for the professional athlete. Continuous intense exercise training may also increase micronutrient requirements, either by increasing frequency of use or by increasing losses from the body which resulting in the need for an increased dietary intake [8].

This cross sectional study was designed to assess the dietary diversity, dietary habits of the athletes and to determine the anemia, Iron, Folate, Vitamin B_12_ and complete blood count (CBC) level, which include RBC count, Hb, status of the Ethiopian professional distance runners using dietary and biochemical method of nutritional assessment.

The biomarkers which used for good indicator of body Iron, Folate and Vitamin B_12_ statuses of the athletes such as transferin, ferritin, serum-folate and serum-vitamin B_12_ were analyzed. In addition to these, acid glycoprotein (α-1AGP) parameter; which is an acute phase proteins or inflammatory biomarker was conducted.

## Methods

### Study subjects and site description

The field work was carried out in Addis Ababa national stadium in the period of February to April 2014. Addis Ababa is the capital city of Ethiopia and it is the largest city in the country. The city has a total population of nearly 4 million [9] with an area of 540 square kilometers (54,000 hectares) and it is sub-divided into ten sub-cities and hundred kebele administrations [10].

It has an altitude range of 2200 to 3000 m above sea level, average temperature of 22.8 °C and average rainfall of 1180.4 mm [11]. The national stadium is found at the heart of the capital city in the way of down town. The study subjects were male and female professional athletes (distance runners) of age range with 17–35 years, who registered and doing continuous training under the Ethiopian Athletics Federation and with their respective coaches.

### Study design

The study design was a cross-sectional method with socio-demographic, food frequency, dietary diversity and biochemical method of hematological and micronutrient status assessment of 101 Ethiopian national professional distance runners from short, middle, 3000 m steeple cheese, long and marathon running. Prior to the commencement of the data collection, the research proposal was submitted and presented to the Ethiopian Public Health Institute’s (EPHI) scientific staffs. After intensive discussion the scientific and ethical review office (SERO) of the institute given the ethical approval.

Then following the clearance, the Ethiopian athletics federation, the couches and the athletes informed about the study objective and its importance. Detail written consents and their agreement to participate in the study was approved by signature on the prepared consent form. Then accordingly, they were asked structured and open ended questionnaires for socio-demographic, dietary diversity and food frequency questioner (FFQ) assessment by nutritionists and finally they had given haematological samples for phlebotomist. Recently archived athletes’ performance data was obtained from the Ethiopian athletics federation, by informing the research participant athletes.

#### Dietary diversity data collection

Assessment of the dietary diversity was done using food frequency questionnaire (FFQ) method of assessment according to the method described by Liu [12]. Individual dietary diversity (DD) method of assessment was done based on the method used by Kennedy [13] and these methods were used by adapting to the local context and the result scored according to method used by Ajani [14].

#### Whole blood collection

The blood specimen was collected by well experienced phlebotomist. The fasting blood samples were taken from the anti-cubital vein of the arm, after proper antisepsis with alcohol and sterile cotton swabs. The syringe was connected with two vacuumed tubes which were done one after the other. For complete blood count (CBC) analysis, blood collection was performed in anti-coagulated venipuncture device (blue cap tube with K_2_-EDTA anticoagulant). All the medical equipment used for blood collections were safe and sterile.

#### Serum sample collection

For micro nutrient and inflammatory biomarkers analysis, a serum was separated from whole blood after 30 min of blood collection by centrifugation at 3000 rpm. The tubes used for serum collection were anticoagulant free (red cap tube). Until analysis, the serum was stored in a deep freezer, at a temperature of −70c^°^ at EPHI clinical chemistry laboratory. From sample collection to analysis cold chain was kept by holding the samples in an ice box during transport and putting them into a deep freezer when reaching the clinical chemistry laboratory. All biological samples were transported and analyzed at EPHI clinical chemistry laboratory.

#### Performance data collection

To investigate either hematological variables or micronutrient status or both were associated with athletic performance; the 2013 and 2014 year best time athletes’ record was obtained from the Ethiopian athletics federation (EAF) for each distance categories. The 2013 and 2014 year athlete’s best speed in their particular running distance category was taken as performance measurement. If an athlete has different best time in 2013 & 2014 years, so the average of the two year performance data was used, otherwise his/her resent best time record was used for biochemical comparison. From all running distance, the top 3 best athletes were sorted out and then categorized together as high performer and the last low speed three athletes selected and categorized as low performer. Finally their hematological variables and micronutrient (MN) status were categorized accordingly, then after AVOVA analysis was run using SPSS in order to find if there is any significant association (*p* < 0.05) with their corresponding biochemical status.

### Laboratory analysis

#### Analysis of hematological indices (CBC)

The collected blood samples were analyzed immediately for complete blood counting, which included MCV, MCH, MCHC, RBC count, Hb level, red cell distribution width (RDW) and lemphocytes. The analysis was performed using a hematological analyzer (SYSMEX XT-1800i, ®sysmex corporation, Japan). Hb, RBC and other hematological indices were analyzed before eight hour of blood collection, as the standard hematological sample analysis and the EPHI laboratory analysis protocol.

#### Anemia status assessment method

An altitude adjusted hemoglobin was used to indicate whether high prevalence of anemia present in the athletic population or not, according to the WHO altitude adjusted Hb formula and the Hb cut off points to define anemia [15]. In the study altitude adjustment was used for an altitude range of 2250 m to 2750 m, which is the commonly used training place were in Addis Ababa stadium (~2400 m) and “*Entoto*” >2600 m above sea level.

#### Iron, folate, vitamin B_12_ and AGP assay 

Transferrin and AGP were measured by Cobas Intgera 400 plus Roche Diagnostics, GmbH, Germany). A fully automated instrument which uses immuno-turbidity-meteric method of analysis. However the Serum-ferritin, folate, and vitamin B_12_ were measured using Elecys 2010, Roche diagnostics, GmbH, Germany, an analyzer which uses Electro-chemiluminescence immunoassay “ECLIA” method of analysis.

#### Iron, folate, vitamin B_12_ and AGP cut offs

The body iron status was assessed by measuring serum ferritin and transferin biomarkers according to WHO guideline [5, 16]. According to the guideline, ferritin of (<12 μg/L) considered as iron deficient and ferritin of >200 μg/L is considered as iron overload. Serum-folate was analyzed as an indicator for the folate status of the athletes. According to the WHO folate cut off points reported in 2012, a serum folate concentration of >20 ng/ml is considered elevated, 6–20 ng/ml is normal, 3–5.9 ng/ml is defined as moderate deficiency and concentration of <3 ng/ml is considered presence of severe folate deficiency [17].

Vitamin B_12_ status was assessed by measuring serum cobalamin level according to method developed by WHO [18, 19]. Serum vitamin B_12_ is considered severely deficient when it is <150 pg/ml, moderately deficient when b/n 150–200 pg/ml range, and normal when it is >201 pg/ml [20]. The lower limit normal range for vitamin B_12_ is 200–250 pg/ml but it hasn’t internationally agreed upper normal range [6]. The presence of disease or inflammation can be determined by measuring C-reactive protein (CRP) and acid glycoprotein (α1-AGP), as they measure the rise of acute phase proteins due to inflammation. In this study AGP was used to assess the disease/inflammation status of the athletes.

### Statistical analyses

All data collected with questionnaires for food frequency, dietary diversity, health & exercise related questions and the data obtained from biological sample analysis were fed to SPSS software version 20 for all descriptive and ANOVA analysis. And the analyzed statistical results were compared using their counterpart reference ranges, to decide whether they are in normal range, mild, moderate or severely deficient and also to find if any significant differences (*p* < 0.05).

However before going to statistical analysis every socio-demographic and hematological data’s were checked for their normal distribution. Given that the serum-ferritin, folate and vitamin B_12_ data was skewed and so, not met the assumptions of normality (*p*-value < 0.05 for Shapiro-wilk test), the data was log transformed then statistical analysis was done and the back transformed mean was used for inferential statistics. However AGP, RBC, Hb, and transferin data was fit for normality assumption for Shapiro-wilk test, so non-transformed data were used to find significant differences and to make inferential statistics.

## Results

### Exercise related and socio demographic characteristics of the athletes

As the data shown in Table 1 below; the mean age (±Sd) of the athletes was 21.3 ± 3.2 year. And 71.3 % of the athletes were in the range of 19–25 years, nineteen athletes (18.8 %) were in the range of 17–18 years, and the rest (9.9 %) were in the range of 26–35 years of age. Sixty eight athletes (67.3 %) were orthodox by religion, 9.9 % were Muslim, and 21.8 % were protestant by religion. In this study, the largest ethnic group was Oromo (41.6 %) followed by Amhara (25.7 %) and Tigrie (12.9 %) but the rest were a collective of other ethnic groups. From 101 athletes, only two athletes (2 %) were married, all the rest were single. Regarding the intensity of their exercise; most of the athletes, (78 %) had 6 days training per week for 2 h of exercise per days, 8 % of them had 5 days training, 7 & 6 % of them had 3–4 days and 7 days training respectively per week for the same exercise hours.

**Table 1:** Socio demographic and exercise related characteristics

Variables	Frequency	Percent
Sport category and sex		
Marathon runners		
Male	10	9.9
Female	6	6
Long (5 &10 km) runners		
Male	8	8
Female	10	9.9
steeplechase (3 km)		
Male	9	9
Female	5	5
Middle distance (800 m)		
Male	12	11.8
Female	10	9.9
Short (100–400 m)		
Male	18	17.8
Female	13	12.9
Weekly training days		
7 days per week	6	6
6 days per week	79	78
5 days per week	8	8
3–4 days per week	7	7
Training hour per day		
1:30–1:45 h	44	43.56
2:00–2:30 h	57	56.44
Sleeping hour per day		
> 12 h	4	4
11–12 h	29	28.7
9–10 h	50	49.5
7–8 h	16	15.8
6 or less Hrs	2	2
Training place		
Stadium, sululta, gefersa, holeta.	28	27.7
Stadium, sebeta,Ararat, CMC.	26	25.7
Stadium, entoto, mekele, A.kality.	32	31.7
Mixture of all	15	14.8
Athletes age range		
17–18	19	18.8
19–25	72	71.3
26–35	10	9.9
Religion		
Orthodox	68	67.3
Muslim	10	9.9
Protestant*	22	21.8
Others	1	1
Ethnicity		
Oromo	42	41.6
Amahara	26	25.7
Tigre	13	12.9
Others**	20	19.8
Marital status		
Single	99	98
Married	2	2
Divorced	0	0
Widowed	0	0
Sex of athletes		
Male	57	56.4
Female	44	43.6
Average monthly income (ETB)		
≤ 1000	9	9
1001–1500	24	23.8
1501–1999	47	46.5
2000–2500	17	16.9
2501–3500	4	4

*Hawariat; **Welaita, Sidama, Gurague, Harari, Hadya, Hamer & Benishangul

### Dietary diversity of the athletes

The data in the Table 2 shows that, the most consumed food groups in 24 Hour period were; cereal/grain products, vegetables, fruits and fats & oils. But the least consumed food types were fish and organ meats. As we can see in the table; 60.9 % of the participant athletes lie in medium dietary diversity followed by 36.1 % in the range of low diversity and 3.3 % fall in high diversity range. The Dietary Diversity Score (DDS) of individual subjects ranged from 2 to 10. And the overall mean DDS among subjects were 5.44 with a standard deviation of 1.8.

**Table 2:** Food groups consumed in 24-hour dietary recall and their dietary diversity scores

	Short (%)	Middle (%)	Steeple chase (%)	Long (%)	Marathon (%)	Total
Food groups						
Cereals	90.3	86.4	100	100	100	95.3
Milk and milk products	8.7	40.9	42.9	11.1	37.5	34.2
Egg	32.3	31.8	42.9	27.8	50.0	36.9
Legumes, nuts and seeds	32.3	59.1	50.0	22.2	62.5	45.2
Other vegetables	68.9	67.8	61.2	63.1	65.7	65.3
Vitamin A rich fruits	48.4	81.8	50.0	61.1	62.5	60.8
Fats and oils	69.8	73.2	75.1	72.9	81.1	74.4
Organ meat	3.2	0	0	0	6.3	1.9
Flesh meat	38.7	40.9	42.9	11.1	37.5	34.2
Dark green leafy vegetables	38.7	27.3	57.1	33.3	37.5	38.8
Fish	3.2	0.0	0.0	5.6	0.0	1.8
Other fruits	52.4	84	53	64	65.5	63.8
White roots and tubers	58.1	45.5	71.4	61.1	62.5	59.7
Vitamin A rich vegetables	58	62	55.3	61	59	59.1
Beverages	58.1	22.7	50.0	33.3	31.3	39.1
Dietary diversity terciles						
Low (1 to 4)	38.71	40.92	14.3	55.55	31.25	36.1
Medium (5 to 9)	58.1	54.6	78.6	44.45	68.75	60.9
High (10 to 14)	3.23	0	7.14	0	6.25	3.3

### RBC and hemoglobin level of athletes

The mean ± Sd of crude and altitude adjusted Hb of male athletes were 16.7 ± 0.82 and 15.4 ± 0.82 g/dL and for females were 15 ± 0.88 and 13.7 ± 0.88 g/dL respectively. The mean ± Sd of RBC count for male and female athletes were 5.45 ± 0.34 Vs 4.85 ± 0.35 RBC count × 10^12^ respectively. There was statistically significant correlation observed between Hb and RBC count (*p* < 0.001). The same way as in Hb, there was significant difference (*p* < 0.01**)** between the male and female RBC level.

### Anemia status of athletes based on Hb cut offs

According to mentioned cut off points for Hb in the method section; the percentage of athlete which were anemic (Hb < 12 g/dL) was 3 %, and among them the two athletes i.e. one male and one female runner were from long distance (5 and 10 km) running while the rest one male was from marathon running.

### Iron, folate & vitamin B_12_ status of the athletes

The non-log-transformed mean and Sd of ferritin level for male (n = 57) and female (n = 44) athletes were 134.1 ± 86.8 Vs 63.9 ± 40.4 μg/L respectively. According to the WHO iron cut offs based on ferritin level, the percentage of iron deficient, ferritin (<12 μg/L) were 2 %. The iron deficient subjects were one male and one female athlete. Percentage of first stage iron depletion (ferritin <50 μg/L, but >30 μg/L) was 22 % and second stage depleted iron store, (ferritin < 30 μg/L, but >12 μg/L) was 13 % and among the 13 % second stage iron deficiency, 11 % were females athletes (Table 3).

**Table 3:** Iron, folate, vitamin B_12_ status of the athletes (x̅ ± sd)

Distance category	N	Sex	RBC count	Hb in g/dl	Ferritin, μg/L)	Transferin (g/L)	Folate, ng/ml	B_12,_ pg/ml
Short (100–400 m)	18	M	5.4 ± 0.4	16.8 ± 0.8	184 ± 65.0	2.4 ± 0.4	7.1 ± 2.2	653 ± 341
	13	F	4.9 ± 0.3	14.7 ± 0.7	65. 7 ± 44	2.6 ± 0.5	8.4 ± 2.9	571 ± 187
Middle (800–1500 m)	10	M	5 ± 0.3	15.3 ± 1.1	50 ± 35	2.8 ± 0.5	8.4 ± 3.1	626 ± 211
	12	F	5.6 ± 0.3	17.2 ± 0.9	89.2 ± 48	2.6 ± 0.4	7.7 ± 2.0	533.5 ± 83.7
Steeple cheese (3000 m)	5	M	4.6 ± 0.1	14.9 ± 0.8	68.6 ± 24.8	2.6 ± 0.4	8.5 ± 1.7	422 ± 239.7
	9	F	5.2 ± 0.3	16.3 ± 0.0	126.3 ± 58.5	2.65 ± 0.5	6.8 ± 2.2	547.3 ± 161.8
Long 5000 & 10,000 m	10	M	4.9 ± 0.4	15 ± 1.0	87 ± 50.3	2.9 ± 0.4	8.4 ± 2.4	472 ± 139.7
	8	F	5.4 ± 0.4	16.5 ± 1.1	150.7 ± 128.2	2.6 ± 0.2	12.8 ± 4.2	615 ± 346.6
Marathon (42 km)	6	M	4.7 ± 0.4	14.9 ± 0.8	41.4 ± 2.4	2.7 ± 0.4	9 ± 5.0	453 ± 117
	10	F	5.5 ± 0.3	16.5 ± 0.8	45 ± 1.7	2.5 ± 0.3	9.7 ± 2.5	541 ± 225
Total	57	M	5.45 ± 0.3	15.4 ± 0.8	134.1 ± 86.8	2.55 ± 3.8	8.4 ± 3.2	586 ± 258
Total	44	F	4.85 ± 0.4	13.7 ± 0.9	63.9 ± 40.4	2.7 ± 0.4	8.5 ± 3.0	528 ± 189
Overall total	101	Tot	5.2 ± 0.5	14.65 ± 1.2	98.9 ± 74.3	2.62 ± 0.4	8.5 ± 3.1	561 ± 231

**Fig. 1:**
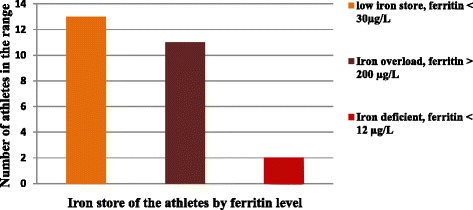
Iron status of professional Ethiopian athletes

In this study the iron overload (ferritin > 200 μg/L) was 11 %, which all of them were male and among all iron overload athletes 8 % were from short distance runners. From the mean ferritin value mentioned above and percentage of second stage iron deficiency indicates, female athletes showed lower iron store than male and this is an indication of more vulnerability than their counterparts. The Fig. 1 below shows the iron status of Ethiopian athletes by their ferritin level.

**Fig. 2:**
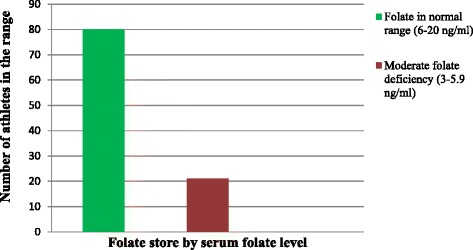
Folate status of the athletes

The mean ± Sd of serum-folate for the total research participant athletes was 8.5 ± 3.1 ng/ml. With a mean values of 8.4 ± 3.2 ng/ml for male and 8.5 ± 3.0 ng/ml for female athletes. According to the above mentioned WHO folate cut off points in 2012, there were 21 athletes (20.8 %) that had moderate folate deficiency, but 80 athletes (79.2 %) had serum-folate within normal range. There was no severe folate deficiency and folate overload case in the study. In addition the proportion of moderate folate deficiency prevalence in both male and female was equal. Figure 2 shows the folate status of athletes.

The non-log-transformed mean and Sd of the serum-cobalamin of the athletes was 561 ± 231 pg/ml. And for the male and female vitamin B_12_ level was 586 ± 258 and 528 ± 189 pg/ml respectively. In the present study, the analyzed vitamin B_12_ data had a minimum value of 210 with a maximum of 1736 pg/ml. According to serum B_12_ cut offs in understanding nutrition [20] all of the athletes were in normal vitamin B_12_ range (vitamin B_12_ > 201 pg/ml), which indicated the absence of severe and moderate deficiency for this nutrient. There was no statistically significant difference observed (p > 0.05) in the mean B_12_ status of male and female athletes and among each running distance categories at 95 % confidence interval.

### Ferritin level by running distance category

When the serum-ferritin data was partitioned by sex, among 44 female athletes 27.3 % had low iron store (<30 μg/L). And this finding is fairly consistent with a study done in Iranian female athletes, which it showed low ferritin level in 33.3 % and iron overload in 12.5 % of the study participants [7]. However, in the current study there was no iron overload female athlete. Distance category has brought significant difference, *P* < 0.05 b/n male ferritin level, but this significance was not observed in female athletes. See Table 4 below the reference section.

**Table 4:** Gender partitioned mean ± Sd of ferritin by running distance

Distance category	Ferritin (male, n = 57)	Ferritin (female, n = 44)
Short distance (100–400 m)	171 ± 1.5^a^	52.0 ± 2.1^a^
Middle distance (800–1500 m)	72 ± 2.2^b^	39.0 ± 2.2^a^
Steeple chase (3 km)	111 ± 1.8^ab^	64.0 ± 1.6^a^
Long distance (5 & 10 km)	124 ± 1.8^ab^	67.6 ± 2.3^a^
Marathon (42.2 km)	65 ± 2.2^b^	41.4 ± 1.1^a^

If any significance (*p*<0.05) observed in ferritin level by distance category either for male or female athletes, so it is superscripted in different letter

### Comparison of the athlete’s serum folate & vitamin B_12_ with references populations

For folate and vitamin B_12_ comparison data see Table 5 below.

**Table 5:** Comparison of the serum folate & vitamin B_12_ with references for various populations

Region	N	Median & (Q1, Q3) Folate in ng/ml	2.5th–97.5th percentile	Region	N	Median and (Q1, Q3) vitamin B_12_ in pg/ml	2.5th–97.5th percentile
Europe	290	9.57	4.6–18.7	Europe	291	357	191–663
Australia	345	10.6	5.3–19.3	USA	178	463	211–946
Ethiopian athletes	101	7.74 (6.3–10.2)	4.2–16.8	Ethiopian athletes	101	516 (431–671)	234–1206
Male athletes	57	7.7 (6.2–10.1)	3.5–17.5	Male athletes	57	520 (434–678)	232–1534
Female athletes	44	7.8 (6.3–10.4)	4.4–17.1	Female athletes	44	508 (398–644)	215–1115

Elecys, 2010 immunoassay analyzer. For the determination of folate and vitamin B_12_ [29, 30]

### Analysis of micro nutrient and hematological variables by sex

There was a significant difference (*P* < 0.05) between male and female Hb, RBC, ferritin and transferin level, however this significance was not observed in folate and vitamin B_12_ status. There was a significant difference in ferritin and folate level by running distance category for male, but the same significance was not observed in female athletes at *P* < 0.05. The rest hematological variables and vitamin B_12_ status were not statistically significant in both male and female by their running distance categories. The analyzed data has shown in Table 6.

**Table 6:** Statistical analysis of micro nutrient and hematological variables by sex

Variation of micro nutrient and hematological variables by sex (Mean ± Sd)						
Sex	RBC	Hb	Ferritin	Transferin	Folate	Vitamin B_12_
Male	5.45 ± 0.34^a^	16.7 ± 0.82^a^	107 ± 2.1^a^	2.6 ± 0.4^a^	7.9 ± 1.45^a^	542 ± 1.5^a^
Female	4.9 ± 0.35^b^	15 ± 0.9^b^	51 ± 2.0^b^	2.7 ± 0.44^b^	8.0 ± 1.4^a^	496 ± 1.4^a^

If any significance observed (*p*<0.05) for specific parameter b/n male and female athletes, so it is superscripted in different letter

### Hematological variables and micronutrient status by their performance

There were 20 male and 20 female professional athletes, who sorted out, since they have recently recorded best time in national and international tournament, and according to their speed, the 10 male and 10 female athletes were grouped in high performer category, as they were the first top three, and the rest ten male and ten female were grouped in low performer category. And then for all the two performer sub category micronutrient and hematological variables were extracted to analyze and find if any relation with the biochemical data. Since equal number of male and females were categorized in each group (high & low performance), the gender difference issue thought to cancel each other. The hematological, micronutrient and performance data were depicted in Table 7 below.

**Table 7:** Association of Athletes’ hematological, micronutrient values and their performance

Hematological variables (Mean ± Sd) of high performer Vs low performer athletes					
Male & female	Hb (g/dL)	RBC count × 10^12^/L	Ferritin (μg/L)	Vitamin B_12_ (pg/ml)	Folate (ng/ml)
High performer	16.2 ± 1.2^a^	5.3 ± 0.14^a^	84.5 ± 63.3^a^	539 ± 153^a^	9.3 ± 3.8^a^
Low performer	15.5 ± 1.0^a^	5.0 ± 0.14^b^	88.6 ± 60.8^a^	487.6 ± 189.5^a^	7.4 ± 3.3^a^

If there is significance (*p*<0.05) for specific parameter among low and high performer, so it is superscripted in different letters

## Discussion

The statistical analysis in the Table 7 shows that even though there was high mean value for the micronutrient, Hb level and RBC of high performer against low performer, but except for RBC values, they were not statistically significant. Irrespective of gender differences, higher performers had higher RBC values than their low performing counterparts (*p* < 0.05).

As mentioned in the method section, the study participants were forty four female and fifty seven male professional Ethiopian athletes (distance runners). The findings of this study show that there was far less anemic and iron deficient athletes as compared to research reported in Korea for distance runners, badminton and shooting athletes in 2007; a study done in Israel at 2004 in male and female top-level basketball players and another study conducted in Ethiopian women of reproductive age in 2010 [20, 21, 4]but it is relatively higher as compared to a study reported in iron status of experienced male and female distance runners in Canada; A study conducted in Germany to investigate the relationship between hematological indices, iron status of athletes and their performance; and also a study done in Spain to investigate the iron status of Spanish junior soccer and basketball players [22–24].

As the detail health related interview and AGP parameters showed that presence of inflammation or infection, parasitic infestation in athletes were very lower than some reports for the general population in the country. Most of the female athletes participated in this study were single and thus, factors that could lead to iron deficiency such as birth spacing, pregnancy and lactation were over-ruled. The was frequent high consumption of cereals like “*teff*” in complete fermented form called “*Injera*” and barely in its partly fermented and non-fermented form in *Beso* and *Chiko* [25] low consumption of beverages like coffee and coca cola; all the above mentioned factors thought to contribute their parts to decrease the high micronutrient deficiency prevalence in the present study.

In the current study, the prevalence of moderate folic acid deficiency (folate <5.9 ng/ml) was 20.8 %; however, there was no athlete with severe folate deficiency (<3 ng/ml). This finding was in line with reports from folate status for recreational endurance athletes in Germany [26], which showed a 15 % prevalence of folate deficiency (<5.38 ng/mL) and 14.9 % in the present study.

The vitamin B_12_ data had a minimum value of 210 pg/ml. Based on the cut off ranges used, all of the study participant athletes were in the acceptable range (B_12_ > 201 pg/ml) which indicate the absence of severe, moderate or mild deficiency for this nutrient. And this present study was inconsistent with a study done on vitamin B_12_ level of recreational endurance Athletes in Germany and other studies have been done on healthy sedentary population in different parts of the world [26–28] as there is relatively high vitamin B_12_ status observed in the participant athlete’s than ever reported.

There was statistical significant difference observed b/n male and female athletes in their red blood cell, hemoglobin and ferritin; which all these are expected naturally from biological and physiological gender variation, however this difference was not observed for folate and vitamin B_12._ This finding agrees with a study conducted in Jordan with absence of differences in vitamin B_12_ status of among 20–40 years old healthy male and female individuals [27]. In addition there is no sex dependent cut off points for Folate and vitamin B_12_ by WHO, NIH, CDC and other worldwide health organizations. Running distance category has brought significant difference in their ferritin and folate level for male but not in female athletes at 95 % confidence interval.

The ferritin level of male marathon runners was lower than short male distance runners which can prove that the iron demand of marathon runners is higher than short distance runners. The absence of significant difference in ferritin status of male long distance running and steeple chase runners can be explained by; running for nearly the same running distance and presence of additional barrier in steeple chase can equalize the iron demand despite of their exercise intensity and dietary patterns.

However middle distance runners showed significantly lower mean ferritin level; compared to steeple chase and long distance is not expected, which may be happen due to presence of high exercise volume and low dietary iron consumption. Exercise volume and exercise intensity of the athletes were very subjective and it was largely relied up on the athletes and their coaches. Diet preference and amount of consumption also can matters, as this study measured the frequency and diversity of athlete’s food consumption, so it is difficult to associate the dietary intake with their ferritin level. Even though there was the same trend in mean ferritin variation was observed from short to marathon runners in female athletes as that of male, but none of them were statistically significant in female ferritin level at *p* < 0.05.

### Strength and limitation of the study

This study has assessed the dietary diversity, food frequency, hematological variables (CBC) and micronutrients status such as iron, folate, and vitamin B_12_ of the athletes and try to associate with their performance of Ethiopian professional athletes. As there was no similar previous study related with these athletes, so the study could fill the existing research gaps and could serve as a base line research data.

The major constraints of this study were absence of enough budgets due to high cost of reagents, scarcity of national base line data, shortage of reference value for the biochemical parameters and absence of related research works; however passing all these challenges the study came to the ground. In the contrary the study have limitations and possible weakness; such as parameters like RBC-folate, C-reactive protein, transferin saturation or total iron binding capacity (TIBC) are not included because of absence of reagents during the study. In addition rather of using the crosses sectional study ; time series data may be more convenient for better analysis of cause and effect relationship.

## Conclusions

Monitoring the diet (macro, micro-nutrient and fluid intake) of the professional athletes is very crucial to balance their day to day demand and also to substitute losses from strenuous exercise, for repairing injured tissues and to maximize flexibility. The present study assessed the most important micronutrients status such as iron, folate, vitamin B_12_ and hematological status which are very known to have tremendous effect on athlete’s health, performance and endurance. Although the high mean micronutrients level and hematological values  shown in high performer athletes, however none of them were statistically significant and only the RBC of high performer athletes was significantly (*p* < 0.05) associated with performance. In contrast to other trace elements assessed, there was no vitamin B_12_ deficiency, furthermore the result showed higher vitamin B_12_ status compared to the other research findings reported so far.

Given, 11 % of the athletes have serum ferritin >200 μg/L which indicates presence of iron overload. Therefore, even though it is common to take dietary supplements such as iron-folate, multivitamins and other trace minerals in the athletes suspecting micro nutrient deficiency; however they should be prescribed based on clinical testing for possible consequences of iron overload and trace element toxicity. Future studies should investigate the reason behind the observed high RBC in high performing athletes. Such study findings may need to be repeated in very super athletes from the Kenyans’ and Ethiopian and also for its consistency in other parts of the world.
